# Follow-up investigation of ethics approvals and regulatory compliance in publications from the IHU-Mi

**DOI:** 10.1186/s41073-026-00234-x

**Published:** 2026-08-12

**Authors:** Fabrice Frank, Elisabeth M. Bik, Gideon Meyerowitz-Katz, Jérôme Barriere, Éric Billy, Véronique Saada, Alexander Samuel, Jacques Robert, Lonni Besançon

**Affiliations:** 1 Independant Researcher, Essaouira, Marocco; 2 Harbers Bik LLC, San Francisco, USA; 3 University of Wollongong, Wollongong, Australia; 4 Medical Oncology Department, Polyclinique Saint-Jean, Cagnes-Sur-Mer, France; 5 Independant Researcher, Strasbourg, France; 6 Biopathology department, Gustave Roussy Anti-Cancer Center, Villejuif, France; 7 Independant Researcher, Nice, France; 8 INSERM Unité 1312, University of Bordeaux, Bordeaux, France; 9 Linköping University, Linköping, Sweden

## Abstract

**Background:**

Following our previous investigation of 456 articles published by the IHU-Méditerranée Infection (IHU-Mi) institute in Marseille, France, subsequent editorial actions, institutional reviews, and reports from French authorities provided additional evidence relevant to our concerns. We therefore extended our investigation to the broader IHU-Mi bibliography to assess potential breaches of the Declaration of Helsinki, French regulations governing research involving human participants, and the consistency between reported studies and the ethics approvals cited.

**Methods:**

We assessed 2,674 IHU-MI publications identified through PubMed, Google Scholar, PubPeer, and manual searches. We excluded articles not reporting research involving human participants, articles clearly reporting retrospective studies without ethics flaw, and articles for which editorial investigations had clarified our concerns. We compared the remaining articles with the relevant French legal framework and, where available, with ethics approvals obtained through public transparency procedures or other sources.

**Results:**

We identified ethical or legal concerns in 853 articles from this institute. These included at least 75 articles with retrospective approvals, 194 describing research conducted abroad without mention of local approvals, 343 not mentioning ethics approval, and 426 describing sample collection from healthy volunteers with concerns. Among 374 articles for which we obtained the cited ethics approval documents, at least 304, or 81.3%, described sample collections that were not covered by the corresponding approval. Since our previous investigation, editorial and institutional responses have led to 64 retractions and 270 expressions of concern, and have clarified or substantiated many of the concerns raised. We also found that the local ethics committee issuing most of the approvals may not have been sufficiently independent from the authors.

**Conclusion:**

Institutional reports and editorial decisions have substantiated many of the concerns identified in our analysis, although a small subset of cases was attributable to unintentional errors. Our findings indicate widespread and longstanding problems in research ethics compliance within the IHU-Mi bibliography and support the need for continued editorial and institutional review.

**Supplementary Information:**

The online version contains supplementary material available at 10.1186/s41073-026-00234-x.

## Introduction

The regulation of clinical and biomedical research has been a major concern since the twentieth century, particularly in response to the atrocities committed in the name of science on human subjects [1]. In addition to the Declaration of Helsinki [2], most countries have developed thorough legal frameworks to ensure that strict ethical processes are followed in studies involving human participants. In the context of the current study, French legislation is particularly relevant. It classifies research involving human participants into two categories. “Recherche N’Impliquant pas la Personne Humaine” i.e. “Research Not Involving Human Subjects” (RNIPH) involves only the analysis of data or samples obtained during standard care. On the other hand, if sampling or treatment differs from the standard of care, French law designates it as “Recherche Impliquant la Personne Humaine” i.e. “Research Involving Human Subjects” (RIPH). Thus, for example, a study carried out on healthy volunteers or sampling outside of the standard of care is considered RIPH, unless determined otherwise.

France enacted the first law on biomedical research in 1988, known as the Huriet-Sérusclat law. It was amended in 2004 to comply with European regulations. It was later replaced in 2016 by the Jardé law currently in effect. These laws are, overall, similar: They require approval by a CPP (Comité de Protection des Personnes i.e. Committee for the Protection of Persons) formerly called CPPRB (Comité de Protection des Personnes dans la Recherche Biomédicale i.e., Committee for the Protection of Persons in Biomedical Research) before starting any study qualified as RIPH.

Unlike institutional ethics committees or institutional review boards (IRBs) used in many other countries, CPPs are independent regional committees that are separate from the institutions conducting the research. Consequently, approval by a local institutional ethics committee does not necessarily demonstrate compliance with French law for studies involving human participants. In addition, the Declaration of Helsinki requires that ethical review be conducted by a committee independent of the researchers carrying out the study.

In France, RIPH is divided into three categories according to the level of risk imposed on participants, ranging from category 3 (no risk, for instance stool, sputum, or urine collection), 2 (minimal risk, for instance blood or nasopharyngeal sample collection) and 1 (important risk, e.g., a therapeutic trial). The sampling types are classified according to ministerial decrees. A CPP approval is given for a specific study design and any change to this design requires a new approval. A single approved study may give rise to several publications, but those publications must remain within the scope of the approved study design. RIPH1 also requires an approval from ANSM (Agence Nationale de Sécurité du Médicament i.e., National Agency for the Safety of Medicines).

Here, we investigate publications from the Institut Hospitalo-Universitaire Méditerranée Infection (IHU-Mi), a large clinical and biomedical research center in Marseille in the south of France. During most of the period covered by this study, the institute operated in close association with Aix-Marseille University (AMU), the university responsible for a substantial part of its academic oversight and research governance. The IHU-Mi has been the subject of international research controversy since the advent of the COVID-19 pandemic, because the institute prominently promoted chloroquine and hydroxychloroquine as treatments for COVID-19. The institute’s most influential paper on the topic was published in March 2020, cited more than 6,000 times (according to Google Scholar), and retracted in 2024 [3] for both methodological and ethical concerns [4], in addition to issues with the peer-review process. This controversy led to our previous systematic investigation of 456 IHU-Mi research publications [5]. That study identified numerous concerns related to ethics approvals and regulatory compliance.

In this paper, we report a broader investigation of the IHU-Mi bibliography, examining the adequacy of ethics approvals cited in published studies and incorporating evidence from subsequent editorial actions, institutional reports, and recovered ethics approval documents.

## Methods

All numbers reported in this manuscript are current as of April 12th 2026. All underlying data are available in the supplementary materials. In this manuscript, we use the term "ethics approval" for any document cited by authors as evidence that a study involving human participants underwent ethical review. Depending on the period, country, and study design, such approvals may have been issued by a CPP, an IRB, or another ethics committee. When relevant, we distinguish between legally required regional CPP approvals and approvals issued by local institutional ethics committees. In the present case, many of the approvals examined were issued by a local ethics committee operating within the IHU-Mi rather than by an independent CPP.

### Cohort inclusion

A cohort of 2,674 publications from the IHU-Mi was identified using Google Scholar and PubMed. Because PubMed limits search results to 1,000 entries, we used multiple search queries, each excluding specific authors (e.g., “-XXX”, where XXX is an author name) and then merged the resulting records. Some articles not indexed in PubMed were only accessible through Google Scholar or other sources, including PubPeer.

First, we excluded articles that did not report research involving human participants. This left 1,807 articles for further analysis.

Second, the ethics statement of each article was assessed according to two criteria:

- Compliance with French law applicable at the time the study described in the article was conducted.
- Compliance with the Declaration of Helsinki and consistency between the ethics approval cited and the research reported in the paper.

**Fig. 1:**
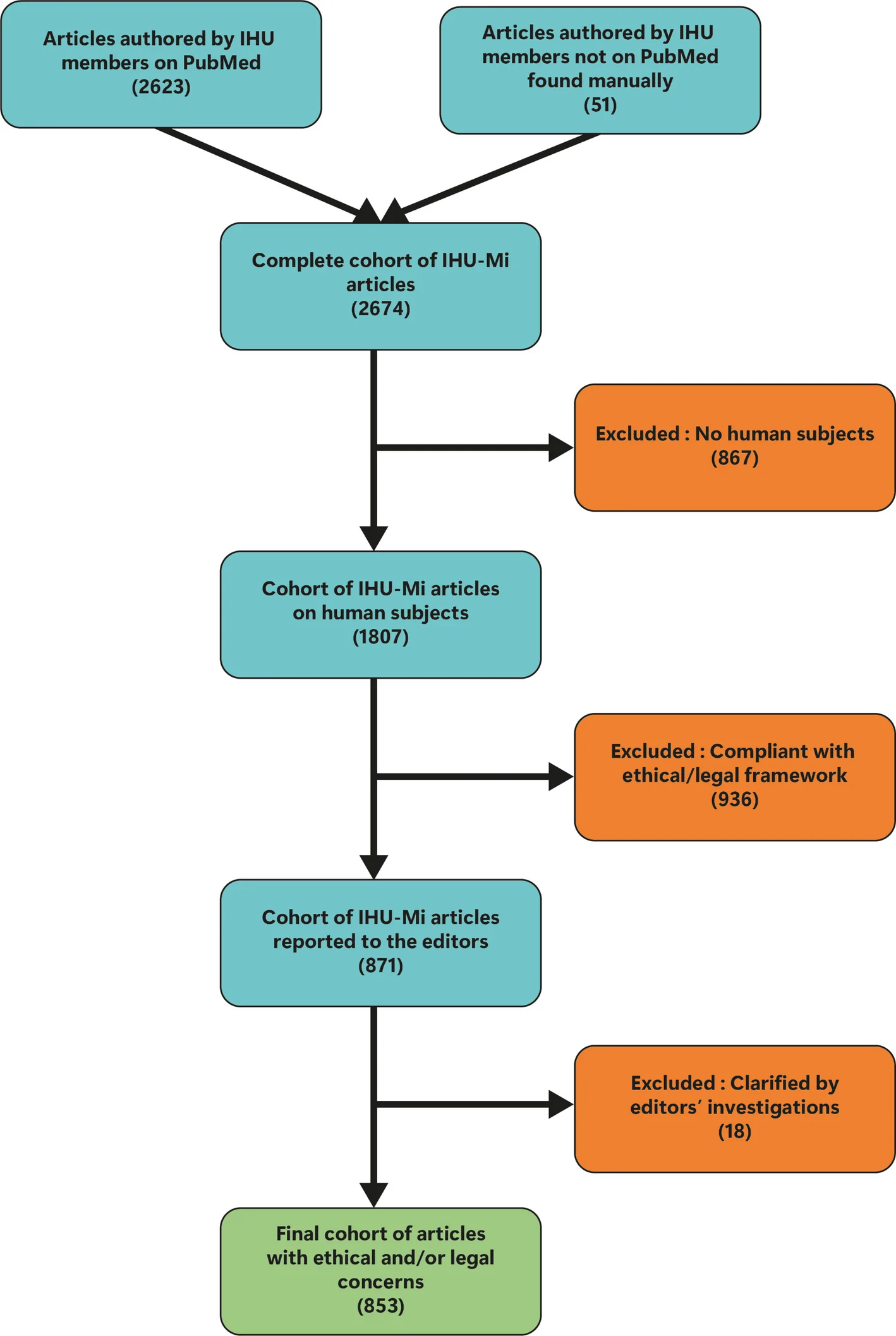
Articles inclusion flowchart

Articles reporting studies found to be compliant with both criteria were excluded from further analysis. At this stage, the cohort comprised 871 human research articles that appeared to be non-compliant with French law or ethics requirement. All of these articles were reported to the editors of the journals in which they were published. While many cases remain under investigation, in some instances editorial teams clarified the concerns and concluded that the articles were not problematic. In 18 such cases, our own final analysis aligned with this assessment, and the corresponding articles were excluded from the set of papers investigated here. Consequently, the final cohort comprised 853 articles. The inclusion flowchart is shown in Fig. 1.

The emails sent to editors varied in type. An initial batch, described in our previous article^5^, consisted of template emails. Subsequent communications were tailored to outline specific concerns in greater detail. Follow-up emails were also sent when prompted by institutional reports or editorial decisions. All articles identified as potentially problematic were reported to the relevant journal editors. Emails were sent between December 2021 and April 2026, and additional correspondence may occur after the completion of the investigation and analysis presented here.

In several cases, there may be legitimate explanations for the apparent violations identified. Where articles provided clear descriptions of the study design, it was usually possible to ascertain whether they complied with the applicable legislation and ethical framework. In other cases, incomplete descriptions of the study design did not allow us either to establish a clear violation or to dismiss our concerns. In some articles, the sampling date was not reported, making it impossible to determine which French law applied. As these articles required further investigation, they were included in the cohort of articles with unresolved concerns pending editorial review.

For clarity, we refer throughout this study to the 2024 version of the Declaration of Helsinki. However, because the articles investigated here were published between 1994 and 2025, it is important to clarify that the following relevant provisions cited in the Results section and in Table 1 were already present in the 1989 version of the Declaration, although article numbering has changed over time:

- Article 10 of the 2024 version was already present in the introduction of the 1989 version. This article requires that medical research involving human participants be conducted in accordance with applicable national laws and regulations, in addition to international ethical standards.
- Article 23 of the 2024 version was included in article 12 of the 1989 version. This article requires ethics committee approval before the start of research involving human participants and further requires that such review be conducted by a committee independent of the researchers.
- Article 27 of the 2024 version was article 10 of the 1989 version. This article requires particular caution when enrolling participants who are in a dependent relationship with the researchers and recommends that informed consent be obtained by an appropriately qualified individual who is independent of that relationship.

This study was not registered and our analysis was not pre-registered. For privacy purposes, we decided not to publish our communications with the editors at this time, but these are available upon reasonable request.

### Ethics approval recovery

A total of 68 different ethics approvals were cited across the 853 articles included in our cohort. We sought to obtain these approval documents in order to assess compliance with the applicable ethical and legal frameworks. Some of these documents were obtained through the French transparency procedure (CADA: Commission d’Accès aux Documents Administratifs i.e. Commission for Access to Administrative Documents), which guarantees public access to administrative records. In short, we first contacted the institution directly to request access to the relevant ethics approvals. After one month without a response, we appealed to the CADA, which issued an opinion stating that the ethics approvals should be made public and instructed the institution to release the documents. Some approval documents were published online by ANSM after a CADA request [6], while others were obtained following email requests to the new leadership of the IHU-Mi or to the affiliated AMU hospital.

Our primary goal was to obtain several ethics approval documents frequently cited in the IHU-Mi bibliography and previously reported in our earlier investigation. Concerns regarding these approvals had already been communicated to the relevant editors and publishers. Despite the CADA opinion, the IHU-Mi did not accept to provide several of the requested ethics approvals. Given the difficulty of obtaining them, we focused our efforts on the approvals most frequently in published articles.

### Final assessments

Different evaluation methods were used to assess the legal and ethical concerns identified in the IHU-Mi articles. Concerns were raised through a variety of mechanisms, including PubPeer reports, author disclosures, and our previous analysis. In some cases, these were double-checked by two independent parties, when possible, but due to the very large number of concerns not all issues could be double-checked. Many concerns were straightforward and did not require independent review; the issue was sometimes simply the absence of any reported ethics approval. When discrepancies arose between two evaluations, the less severe assessment was retained.

The first step consisted of a legal evaluation, followed by a comparison between the study design described in the ethics approval document cited in the article and the study design described in the methods section of the publication. In some cases, the article appeared inconsistent with the cited ethics approval. In some cases, it appeared non-compliant with French law. In some instances, both concerns were identified. Finally, a subset of articles required further investigation to confirm or dismiss concerns. We restricted our analyses to cases for which sufficient information was available to support a reliable assessment. Consequently, the numbers reported in the Results section should be regarded as conservative estimates and may increase if additional information becomes available for currently unresolved cases.

Approvals issued outside of France were not assessed in the present study, with regard to their validity or relationship to the research described in the publications.

## Results

We assessed 853 IHU-Mi articles that met our inclusion criteria and identified multiple categories of ethical and regulatory concerns. We obtained 23 of the 68 ethics approval documents cited in these articles [7] through the CADA procedure described in the Methods. Because approvals issued by the IHU-Mi ethics committee were frequently reused across multiple studies, these 23 approval documents corresponded to 374 IHU-Mi publications.

For example, approval 09–022 was mentioned in 248 articles at the time of our previous investigation. Despite publication of our findings, it continued to be cited in subsequently published articles. At the time of this current analysis, approval number 09–022 has been cited in 258 publications, two of which [8, 9] were submitted after the French press wrote about our concerns [10]. Among these 258 articles, 11 have been retracted and 149 are under expression of concern so far.

Classification of concerns was not always straightforward. The studies were conducted over several decades and were therefore subject to different regulatory frameworks. In addition, many articles provided only limited descriptions of study design, participant recruitment, or sample collection. In some cases, key information such as sampling dates or countries of recruitment was not reported, making it difficult to determine which regulations applied.

Our analysis identified:

- 75 articles describing studies for which ethics approval appeared to have been obtained retrospectively;
- 194 articles describing research conducted outside France without mention of local ethics approval;
- 343 articles that did not mention any ethics approval;
- 426 articles reported studies involving healthy volunteers, indicating that the research could not be classified solely as analysis of samples obtained during standard care.

These categories are not mutually exclusive, and individual articles could fall into more than one category.

Among the 374 articles for which we obtained the cited ethics approval documents, at least 304 i.e. 81.3% (304/374) report collections of described sample collection or study procedures that appeared inconsistent with those approvals.

Estimating the number of studies that were clearly non-compliant with French law was difficult, because many articles provide insufficient information to distinguish prospective from retrospective research. Nevertheless, 85 articles explicitly described prospective studies. Under French regulations, such studies generally require CPP approval. Among these, only eight reported a CPP approval, one of which has since been retracted because the approval did not match the reported study design. We therefore estimate that at least 78 articles describe biomedical research that appears not to comply with the applicable French law.

### Institutional reports

Through the CADA process, we also obtained a report commissioned by AMU (Aix-Marseille University) following the advice of the Office Français pour l’Intégrité Scientifique (OFIS), i.e. the French Office for Scientific Integrity) [11]. The AMU report concerned 8 articles and found that 7 of them were not compliant with French law: six [6 out of 8] because of lack of CPP approval, and one [one out of 8] because informed consent had not been collected. The 8th article cited an incorrect ethics committee decision and the authors were unable to provide the correct one; it was therefore not compliant with Article 23 of the Declaration of Helsinki, which requires ethics approval to be obtained before the start of participant recruitment.

Overall, the AMU report on the ethics of 8 articles also states that both the IHU-Mi and the University’s conduct raise concerns regarding governance and ethical review mechanisms. All 8 articles have now been retracted.

Furthermore, an institutional inspection formerly carried out by IGAS (Inspection Générale des Affaires Sociales i.e., General Inspectorate of Social Affairs) showed that the authors were either close to members of the IHU-Mi ethics committee that issued those approvals or were themselves members of that committee [12]. This represents a direct conflict of interest. The IGAS report stated that “*The IHU has its own ethics committee, the composition of which does not sufficiently guarantee its independence and whose working methods do not allow for an informed decision*” and that “*the composition of the ethics committee does not sufficiently guarantee its independence. Unlike the CPPs, and moreover since the random draw implemented since July 2, 2018, the ethics committee is composed of six persons, either close to the IHU or directly interested in the research conducted at the IHU*”, which is inconsistent with article 23 of the Declaration of Helsinki. The IGAS report also explains that “*The working arrangements do not allow for an informed decision by the ethics committee*” and that “*The sheet [used by this committee] does not present the study protocol or the condition of the patients. It is therefore very difficult for the committee members to accurately assess how the study will be conducted*”. This latter observation is consistent with the difficulties we encountered when assessing study designs from the published articles.

The lack of willingness from the university to independently and factually analyze these articles indicates that it is not just the ethical governance which may be problematic. It seems indeed that These findings raise broader questions regarding the university’s governance and oversight mechanisms. In fact, an institutional report from HCERES (Haut Conseil pour l’Evaluation de la Recherche et l’Enseignement Supérieur, i.e., High Council for Evaluation of Research and Higher Education) recommended in May 2025 that the institute and the University take part in the process of the process of auditing their publication record [13].

The HCERES report identified systematic non-compliance with French and European laws governing clinical research involving human participants at the IHU-Mi during the leadership of Professor Raoult. It criticized the institute for failing to follow standard protocols for ethics review, for maintaining inadequate internal ethics structures that were not independent from researchers, and for failing to properly retain documentation of protocols, consent forms, and ethics approvals. The report identified dozens of articles that used human samples without appropriate CPP review or without informed consent, describing them as "*publications that should not have existed in their current form*". These findings were consistent with concerns identified in our analysis.

### Editorial decisions

To date, 64 retractions have occurred and 270 expressions of concern have been issued by various editors and publishers. Later updates of these figures will be found online for retractions [14] and expressions of concern [15].

Important information emerged from retraction notices issued by PLoS journals, which offers insight into the communication between the AMU Ethics Committee and journal editors [16–20]. In several cases, the editor found that the explanations provided by the authors were inconsistent with French law. In other cases, the cited approval was retrospective or unrelated to the research for which it had been used. These findings are consistent with the difficulties in locating the corresponding ethics approval documents, as described in the institutional report discussed above.

In one interesting retraction notice [17], an AMU representative replied to the PLoS editors that “*the authors wrongly describe this study as a prospective study*” and that “*this is a retrospective study instead” that “did not involve the human person*” and required “*no written consents”*. Because these statements appeared inconsistent with the reported data, the editors stated that “*[t]he institute’s response did not resolve the concerns regarding the discrepancy between the information in the article and the information in the ethics approval documentation*”. In another retraction notice, an AMU representative stated that the collection of lice from the bodies of human participants did not constitute research involving human participants, a position that the PLoS editors did not accept [18]. These exchanges are consistent with both the institutional reports described above and the concerns identified in our analysis.

In addition, in all of these retraction notices, the PLoS editors reported a conflict of interest between the internal IHU-Mi ethics committee (which is not the legally required regional CPP) that granted the approval and the authors [16–20]. This concern had also been raised by the IGAS inspection mentioned above [14]. In November 2023, Elsevier issued a Publisher’s Note stating that it had appointed an independent publishing ethics advisor to support the investigation on the articles on which we had raised concerns [21]. Following this announcement, Elsevier issued expressions of concern for 170 IHU-Mi articles. Unfortunately, this investigation remains ongoing.

**Fig. 2:**
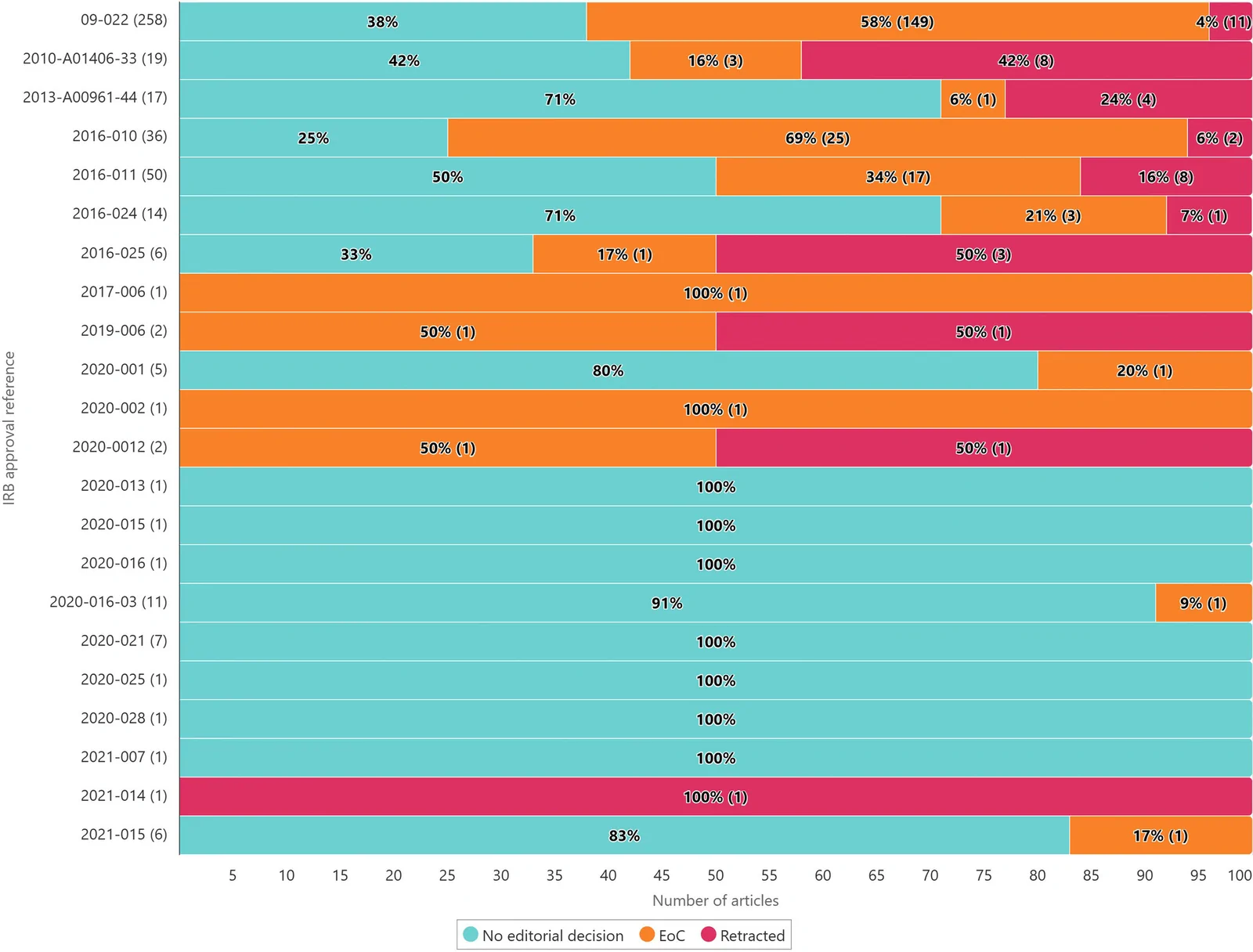
Editorial outcomes following our notifications to journals for articles associated with each ethics approval document in this study. Bars show the proportions of articles with no editorial action, an expression of concern, or a retraction. Numbers in parentheses indicate article counts

Overall, most editorial decisions issued since the publication of our previous article have been consistent with the concerns we reported. The editorial outcomes for articles associated with each ethics approval document obtained in this study are summarized in Fig. 2, showing the proportion of articles with no editorial action, an expression of concern, or a retraction, as per April 2026.

Comparison of ethics approval documents and published studies.

Because approvals issued by the IHU-Mi ethics committee were often reused across multiple studies, the 23 ethics approval documents we obtained corresponded to 374 IHU-Mi publications.

For each approval, we compared the participant populations and sample types described in the approval document with those reported in the studies citing that approval (Table 1). We identified apparent inconsistencies between the scope of an approval document and the research described in 283 of the 374 articles. As per April 2026, 7.2% (27/374) of these articles have been retracted, and 53.5% (200/374) have received an expression of concern.

**Table 1:** Summary of concerns on local ethics approval obtained by the IHU-

IRB approval (With reference, date it was granted on and source of the approval)	Approves	Number of articles found in	Retracted articles	EoC	Concerns
2010-A01406-33 from 2011–01–24 by CPP Sud-Méditerrannée I	Eradication of body lice carrying through permethrin treated clothing: A double blind, randomized versus placebo comparative trial in disadvantaged populations of Marseille	19	8	1	Despite the corresponding study took place between January 2011 and May 2012 and did not mention any samples collection, it was used for various samplings from 2010 to 2019
2013-A00961-44 from 2013–06–24 by CPP Sud-Méditerrannée I	Evaluation of pharyngeal carriage of microorganisms responsible for transmissible acute respiratory infections in Hajj pilgrims	17	3	0	Used for sample collection on healthy volunteers recruited in, travel agencies from 2010 to 2018. Such study requires a CPP approval. Retractions from studies carried out from 2013 also suggests that the protocol we could not reach did not mention all these years
2016–010 from 2016–09–21 by IHU ethics committee	samples of the digestive tract, subject to details (Number of patients, indication for endoscopy) that were never given and does not mention specific samples	33	2	25	14 mention stool collection, 1 mentions oral samples and one urine analysis, seemingly not consistent with collection via endoscopy
2016–011 from 2016–09–21 by IHU ethics committee	stool collection and does not mention specific samples	39	8	16	1 is prospective and involves clinical data collection and approval is mentioned retrospectively, 2 are related to dental plaque, 2 report saliva collection, 2 report oral sampling, 2 report sputum collection, one does not mention the type of samples collected, 3 refer to urine collection and 2 report vaginal sampling. 37 of these articles mention healthy volunteers
2016–024 from 2016–10–19 by IHU ethics committee	“Publication of clinical observations during clinical or retrospective studies using a series of more than 1 patient within the IHU” and excluded specific sampling. It clearly mentioned the requirement for a specific request for each study, which has not been done	14	1	3	2 of these articles report blood sample collection, 2 cerebrospinal fluid sampling, 1 an aortic sample, 1 oropharyngeal sampling, 1 osteoarticular sample, 1 surgical drainage, 1 respiratory sample and 1 mediastinal cutaneous fistula swab
2016–025 from 2016–10–19 by IHU ethics committee	“Detection of microorganisms or unknowns in biological samples such as blood, biopsies, respiratory samples and biological fluids sent at the CNR of rickettsiae”, mentions blood, lymph nodes and biopsies samples exclusively carried out for diagnostic testing with no specific sampling, subject to patients informed consent collection	7	3	1	1 mentions Tropheryma Whipplei, 3 tuberculosis, 1 Fastidious Bacteria, 1 about HIV, 1 vascular infection and none mention rickettsiae
2017–006 from 2017–02–20 by IHU ethics committee	“Development of a LAMP for faster diagnosis in Kingella Kingae”, excludes specific sampling, mentions a retrospective study on articular liquids for children diagnosed between 2014 and 2016	1	0	1	7217 Israeli healthy children for Oropharyngeal swabs with retrospective approval
2019–008 from 2019–08-07 by IHU ethics committee	Comparison of peptide profiles of gingival fluid, saliva and dental plaque in patients with periodontitis and patients free of periodontal pathologies by masse MALDI-TOF spectrometry	1	0	0	Opinion claims a CPP approval is required
2019–006 from 2020–04–23 by IHU ethics committee	“BMRSTUD. Travel-related pathologies and acquisition of pathogens and multiresistant bacteria in medical students doing a practical internship outside France” received “Favorable, subject to a request for an opinion from the Ethics Committee (CPP), subject to a favorable opinion from a CPP” which was neither requested nor obtained	2	1	1	Rectal and vaginal swabs on the students at IHU. Thus, informed consent collected by the authors cannot be considered as valid according to the article 27 of the Helsinki declaration. This is clearly an clinical trial that appears inconsistent with regulatory requirements and was stopped by the French administration, for which the authors have issued a document that appears to have been altered [21]
2020–001 from 2020–01–29 by IHU ethics committee	“Determinant of self-medication behaviors in general medicine” and excludes specific sampling	5	0	1	5 different articles, related to the hydroxychloroquine cohort treated in IHU hospital during the COVID-19 pandemic
2020–002 from 2020–01–29 by IHU ethics committee	Isolation of non-cultivable strains from the oral cavity	1	0	1	Used to make a CT-scan study on paucisymptomatic COVID-19 patients which would have required a CPP approval
2020–0012 from 2020–04-01 by IHU ethics committee	Evaluation of scanographic aspects in the diagnosis and assessment of the severity of COVID-19 infections” and excludes specific sampling	2	1	1	Both report a prospective CT-scan study on the hydroxychloroquine cohort including paucisymptomatic patients
2020–013 from 2020–04-03 by IHU ethics committee	“Evaluation of the different therapies proposed in the management of patients with COVID-19/first 1000 patients treated in Marseille”	1	0	0	Prospective clinical trial on hydroxychloroquine that should have received an authorization from a CPP
2020–015 from 2020–04-03 by IHU ethics committee	“Retrospective study of SARS-CoV-2 carriage in precarious populations diagnosed at the IHU”, mentions 3000 patients and excludes specific sampling	1	0	0	Single study that specifies that the patients were recruited in the accommodation centers for nasal sampling, which characterizes a prospective study that should have obtained an approval from a CPP, especially for a vulnerable population
2020–016 from 2020–04–23 by IHU ethics committee	Genomic diversity and evolution of coronavirus (SARS-CoV-2) in France from 309 COVID-19-infected patients	1	0	0	Consent was collected prior to enrollment. This is a prospective study that required a CPP approval
2020–016-03 from 2020–04–23 by IHU ethics committee	“Genomic diversity and evolution of coronavirus (SARS-Cov\SARS-CoV-2) in France from Covid −19-infected patients” and excludes specific sampling	12	0	1	Research carried out on the prospective hydroxychloroquine treated cohort
2020–021 from 2020–05–13 by IHU ethics committee	Real life diagnosis and management of Covid −19 patients: a cohort Study of 3,737 patients, Marseille, France	7	0	0	The mentioned study required a CPP approval. This was also used for studies on Lupus and Cancer patients as well as hypoxia
2020–025 from 2020–06–08 by IHU ethics committee	Retrospective analysis of SARS-CoV-2 epidemic clusters within the Marseille Jewish community diagnosed at the IHU	1	0	0	Studies based on religion are prohibited in France
2020–028 from 2020–08–13 by IHU ethics committee	Pattern of SARS-CoV-2 infection among dependant elderly residents living in retirement homes in Marseille, France, March-June 2020	1	0	0	Retrospective approval for a study that required a CPP approval
2021–007 from 2021–04–07 by IHU ethics committee	“Early outpatient treatment with hydroxychloroquine and azithromycin in COVID-19: A retrospective cohort of 11,071 outpatients”	1	0	0	The treatment not being a standard of care of COVID-19 by this time, this is a prospective study that should have obtained an approval from a CPP
2021–014 from 2021–04–30 by IHU ethics committee	Rapid isothermal amplification detection of buccal SARS-CoV-2 for ambulatory screening of COVID-19	1	1	0	This paper describes a prospective recruitment in February 2020, thus requiring a CPP approval and displaying a retrospective approval
2021–015 from 2021–05–18 by IHU ethics committee	“Retrospective study COVID cases treated at the IHU from March 2020 to May 2021”	5	0	1	4 report the clinical trial on hydroxychloroquine that appears inconsistent with regulatory requirements, and 1 adresses “Olfactory And Gustative Disorders For The Diagnosis Of COVID-19” which is not the subject of the approval, ANSM filed a complaint for illegal clinical trial
09–022 from 2009–12-09 by IHU ethics committee	“Diverse culture of bacteria in human stools” excludes collection of humans samples specific to the research, clearly mentioning samples in the bacteriology lab in Hôpital de la Timone, Marseille	258	11	149	2 with blood sampling, 1 with breast milk collection, 1 with digestive sample, 2 with ileum samples, 3 with left colon samples, 1 with oral samples, 1 with rectal swab, 1 with right and left colon lavage, 3 with right colon lavage, 4 with skin swabs, 4 with sputum, 13 with urine and 23 reporting vaginal swabs Among the 189 mentioning stool collection, we found 100 clearly reporting healthy patients, thus specific sampling, 4 reporting seemingly healthy patients, 46 not reporting informed consent collection or inappropriate consent collection. 124 of these articles reported sampling outside of France. Thus, we consider that 229 of these articles do not report research in line with the given approval Two of these studies were initiated in 2008 and thus present a retrospective approval which is illegal

For example, ethics approval IRB 2016–010 mentions sampling of the digestive tract, whereas studies citing this approval reported collection of stool, urine, and oral samples. Approval 2016–011 covers stool collection, but was cited in papers describing collection of clinical data, dental plaque, saliva, sputum, urine, vaginal, and oral samples. Similarly, approval 2016–025 was granted for rickettsiae diagnosis, but was used in studies involving Tropheryma whipplei, tuberculosis, fastidious bacteria, HIV, and vascular infections. Ethics approval 2017–006 describes a retrospective study and does not include specific sampling procedures. Yet, it was cited in a study involving oropharyngeal swabs collected from 7,217 healthy Israeli children without mentioning local ethics approval. Approval 2016–024 was issued for the publication of clinical observations and did not include sample collection, but was cited in studies describing blood, cerebrospinal fluid, aortic, oropharyngeal, osteoarticular, and respiratory sampling, as well as interventions such as surgical drainage and mediastinal cutaneous fistula swabbing. The IHU-Mi also investigated the genetic diversity of SARS-CoV-2 viruses isolated from COVID-19 patients under approval 2020–016-03, which explicitly excluded specific sampling procedures. These examples illustrate recurring discrepancies between the scope of the approval documents and the participant populations or sample types reported in the corresponding publications.

The largest group of articles involve ethics approval 09–022, which was granted only for in vitro culture of bacteria from previously collected stool samples in the authors’ laboratory, but was subsequently cited in studies involving sample collection from participants in multiple countries, including children and healthy volunteers. Among the 258 articles citing IRB approval 09–022 that we identified through the same procedure (248 had already been identified in our previous article), our analysis of the reported study designs indicated that at least 235 could not be covered by the scope of the approval. For 8 additional articles, the study descriptions were insufficiently detailed to determine whether use of this approval was appropriate. Thus, only 15 articles appear potentially compatible with this approval, although this assessment should ultimately be confirmed by an editorial investigation.

Several cases involve prospective studies that would have required approval by an independent ethics committee (CPP) under French law, but no such approval was identified. This is the case for 2020–0012, which was granted for a study evaluating computed tomography (CT) scans in COVID-19 patients, and explicitly excluded any form of sampling. However, it was cited in a prospective study investigating hydroxychloroquine as a treatment for COVID-19 infection, which would have required CPP and ANSM approval. Similarly, approval 2020–013 was issued for an article reporting a prospective study of hydroxychloroquine treatment in the first 1,000 COVID-19 patients referred to the IHU-Mi without any mention of CPP or ANSM approval. Approval 2021–007 was used for a study involving 11,071 outpatients who received this same treatment, again without CPP nor ANSM approval. Approval 2021–015 raises similar concerns. It covers five studies, one of which falls outside the scope of the approval, while the others would have required CPP approval. Approval 2020–015 also appears to require CPP approval, as it involved nasal swabbing of approximately 3,000 vulnerable patients.

In the case of approval 2019–006, the IHU-Mi provided a document that, according to an ANSM inspection report, differed from the original version [22]. The original document required a favorable opinion from an independent ethics committee (CPP) before the study could begin, whereas the version provided by the IHU-Mi appeared as a definitive approval without such a requirement. The study involved rectal and vaginal swabs collected from students at the IHU-Mi. According to the ANSM report, the study was not conducted in accordance with Article 27 of the Declaration of Helsinki, because informed consent was obtained by researchers from healthy volunteers who were in a subordinate position to them. The ANSM subsequently halted this study through a health police decision [23].

Approval 2020–01 was cited in a clinical trial evaluating hydroxychloroquine and azithromycin treatment in 80 COVID-19 patients [24]. However, the approval document was issued on January 21, 2020 for an epidemiological study entitled "Determinants of self-medication behavior in general medicine”, before the IHU-Mi had begun investigating hydroxychloroquine as a treatment for COVID-19. The approval therefore appears unrelated to the study for which it was cited and does not appear to cover the research reported in the publication.

Two of the ethics approval documents obtained were legitimate CPP approvals, but further analysis indicated that they had been cited in studies unrelated to the research for which the approvals had originally been granted. The first example is CPP approval “2010-A01406-33” [25] which was granted for the study “Effect of permethrin-impregnated underwear on body lice in sheltered homeless persons: a randomized controlled trial”, conducted between January 2011 and May 2012 [26]. However, this approval was also cited in 18 unrelated studies, including a tuberculosis screening study published in 2021. The second case pertains to CPP approval 2013-A00961-44 [27], which was granted in 2013 for a prospective study involving Mecca pilgrims. The study was conducted between 2013 and 2018. Although the approval document we received does not specify an end date, editors requested additional documentation from the authors to justify its use in later studies. According to information communicated by the editors, the authors were unable to provide such documentation, leading to the retraction of several articles. These findings suggest that this approval was also cited for studies that fell outside its original scope.

Approvals given abroad were not checked, in the present study, for their validity and relation to the described research, investigating them, while outside of the scope of our study, might lead to even more concerns being raised or validated.

Of course, the editors of the published articles have been made aware of these issues and we hope that they will be able to take independent actions. To help them analyze these issues, we have set up a website summarizing all of these articles and giving them direct access to the IRB approval and obtained documents [28].

### Data availability

After completing the analysis, we aimed to inform the editors of unethical and potentially illegal papers, so that they could investigate the issues, and take appropriate editorial action. While the legal consequences for violations of the French ethical framework for biomedical research (or those of other countries where some studies were conducted) is beyond our scope, we did contact editors with our analysis to highlight unethical manuscripts that should be retracted for infringing articles 10, 23 and 27 of the Helsinki declaration. We emailed all editors of the journals that published the 853 IHU-Mi articles investigated in our cohort to ensure that they are aware of the issues found in their content.

We also developed a tool to keep track of papers and their publication status according to different metrics: authors, journals, IRB numbers with export capabilities [29]. An export of this file is available as well as on an OSF repository [30]. This tool is made of a list of the papers we have reported and includes the possibility to filter them by status (Retracted, with Eoc), by author, by journal or publisher, by sample type or IRB approval reference, by review duration and other parameters. This is the tool we have used for the reporting made in this article, either directly or through queries in the corresponding database. The tool is also linked directly from our OSF repository.

Noteworthy, in many cases, we also responded to the requests for clarification from editors. Our tool also provides access to the ethics approval documents obtained during this investigation and supporting materials used in the analyses reported here.

## Discussion

We identified a total of 853 IHU-Mi papers with potential ethical or regulatory concerns through PubPeer comments and our own analysis, involving 68 ethics approval numbers cited by the institute. By contacting research ethics personnel at the IHU-Mi and its affiliated AMU, and subsequently through the CADA process, we obtained 24 key documents relevant to this investigation. Of these, 23 were ethics approvals and one was a report commissioned by AMU following advice from the French Office for Scientific Integrity (Office Français pour l’Intégrité Scientifique, OFIS) [12]. Of note, we obtained ethics approval document 09–022, which was reported in 258 studies at the time of this analysis (248 at the time of our initial investigation).

We learned from PLoS retraction notices and institutional reports that concerns had been raised regarding the independence of the local ethics committee responsible for many of the ethics approvals discussed here. We also found instances in which local ethics approvals appeared to have been used in situations where a CPP approval would have been required, as well as cases in which local approvals were cited for research conducted outside of France. We also identified instances in which legitimate CPP approvals were cited in studies that appeared to fall outside of their scope. In one case, an approval-related document appeared to have been altered.

Although the authors did not initially respond to our concerns, two of them later replied via a preprint, which has since been withdrawn due to defamation [31]. They rely on the analysis made by an ethical committee at AMU which had been shown to be led by a person spreading conspiracy theories and anti-vaccine discourse [32]. Unsurprisingly, despite them claiming that out of the only 269 articles they had analyzed, only 2 might contain issues, 11 had been retracted (although we know of 64 retractions already). This ethical committee found 0.7% (2/269) of articles containing issues. This is, we argue, confirming the issue about this committee. This also tends to confirm our findings both on the bibliography of the IHU and on their ethical skills. The authors later replied in another preprint, now also withdrawn, using screenshots from Twitter social media, to allegedly demonstrate our conflicts of interests.

While plagiarism, data fabrication, and falsification are the most widely recognized forms of research misconduct [33], our findings highlight another important category: ethical non-compliance in biomedical research involving human participants. A common argument is that such ethical shortcomings do not necessarily invalidate the scientific findings. However, research involving human participants should not be relied upon unless both its ethical and scientific validity can be independently established. Failure to comply with ethical requirements undermines public trust in research and may ultimately jeopardize participation in future studies.

Major ethical breaches in biomedical research have been documented previously. For example, the Woo Suk Hwang case led to a national scandal, institutional investigations, and recommendations for changes in guidelines [34]. Similarly, the Paolo Macchiarini case highlighted the challenges institutions may face when responding to serious allegations involving prominent researchers [35]. Institutional resistance to allegations of misconduct and criticism from whistleblowers has also been described in France, including during the Jessus affair [36]. Ensuring compliance with ethical requirements is therefore essential, particularly when concerns affect large numbers of publications.

This case also shares similarities with other large-scale misconduct investigations. For example, the Joachim Boldt case involved a comparable volume of publications and required a coordinated response from multiple journals [37]. This was similar to Elsevier’s decision to appoint a dedicated team to investigate the concerns raised in the IHU-Mi bibliography. The Boldt investigation extended over more than a decade, illustrating the considerable time and resources often required to evaluate large numbers of potentially problematic publications.

### Limitations

Our analysis has some limitations. 

We assessed only the ethics approval documents that could be obtained and did not evaluate approvals issued outside France. Our analysis was based on information reported in published articles, institutional reports, editorial correspondence, and approval documents available to us. In some cases, incomplete reporting in the articles prevented definitive conclusions regarding the appropriateness of a cited approval.

The reasons for which we were not able to access all the available references are: lack of time,

Several factors limited our ability to obtain all cited ethics approval documents, including the large number of approvals involved, limited cooperation from the university and the IHU-Mi, and delays associated with possible overloading of the French transparency procedure (CADA) which gives opinions at a very slow pace. For example, some requested approval documents had still not been provided more than one year after the initial request and several months after a favorable CADA opinion had been issued.

In some cases, a definitive assessment remains difficult because the available information is incomplete. However, many of concerns identified in our analysis have subsequently been supported by editorial decisions or regulatory findings.

Given the large size of the IHU-Mi bibliography and the limitations of the search tools used to identify publications, additional articles with concerns may be discovered in the future.

The inadequacy of research institutions in addressing scientific misconduct is well documented, although several efforts have been made to tackle this issue [38]. Offices for research integrity at the federal or national level have emerged since the 1980 s, but concerns have been raised about their effectiveness and resources [39]. Other solutions have been proposed, including independent nonprofit investigative organizations [40]. Institutional authorities may face financial and reputational pressures when responding to allegations of misconduct [41]. Meanwhile, whistleblowers and science critics might be subjected to harassment or legal threats, while students working in laboratories facing allegations of misconduct might suffer serious career consequences [42].

## Conclusion

Through our analysis of the IHU-Mi bibliography, we identified substantial concerns regarding research ethics compliance. From 1994 to 2024, we identified 853 articles with various ethical concerns, including repeated use of ethics approvals, approvals issued by a committee whose independence has been questioned, lack of legally required approvals, retrospective approvals, use of approvals outside of their stated scope, and publications reporting research involving human participants without reported ethics approval.

On a subsample of 374 articles for which we were able to access the ethics approval documents, we estimated that 81% showed a mismatch between the study described in the approval document and the study reported in the publication. Our exchanges with French regulatory authorities provided additional support for the concerns identified in our analysis.

We conclude that serious concerns regarding research ethics compliance persisted at this institute over decades and that continued correction of the affected literature remains necessary. This is consistent with the HCERES report, which recommended that the authors and the university participate in the correction process by contacting editors directly, a recommendation that has not yet been fully implemented. Furthermore, several retraction notices indicate that the authors were unable to provide requested comments or supporting documentation. Some publishers have also indicated that the institute and its host university misrepresented aspects of certain cases during editorial investigations in attempts to prevent retractions.

Despite the limited actions taken by the authors and their institutions, our findings have so far led to 64 retractions and 270 articles being placed under expression of concern. Continued editorial and institutional review will be necessary to ensure that the scientific record is corrected where appropriate.

## Supplementary Information

Supplementary Material 1.

## Data Availability

All data are available on (https://ihu-correction.com) and in the supplementary data. A CSV extract if also available on (https://osf.io/hb2ze). Our communications with the editors are available upon reasonable request.
